# Design Strategies for Multi-Unit Residential Buildings During the Post-pandemic Era in China

**DOI:** 10.3389/fpubh.2021.761614

**Published:** 2021-10-12

**Authors:** Yanqing Xu, Yi-Kai Juan

**Affiliations:** Department of Architecture, National Taiwan University of Science and Technology, Taipei, Taiwan

**Keywords:** design strategies, COVID-19, multi-unit residential buildings (MURBs), post-pandemic era, refined Kano model

## Abstract

**Objective:** The sudden outbreak of COVID-19 has greatly endangered public health and life safety, leading to new changes in people's housing needs. The purpose of this study is to establish design strategies that are suitable for China's Multi-Unit Residential Buildings (MURBs) in the post-pandemic era, and to identify the users' preferences for these strategies.

**Methods:** This study compiles a set of design requirements by means of a literature review and expert interviews. Three hundred ninety-five online and on-site questionnaires, based on the refined Kano model, were distributed to respondents to reveal their preferences for these strategies. The relationship between the different demographic variables, the preferences of design strategies, as well as the housing unit preferences of home-buyers were also verified by means of an actual project.

**Results:** This study summarizes the four dimensions and 26 design strategies of MURBs in China during the post-pandemic era. These strategies are further extracted into 6 highly attractive, 5 high-value-added and 4 critical quality attributes. In terms of demographic variables, males need more social space, and the elderly need less office space and separate bathrooms in the master bedroom. Due to the impact of the epidemic, people with higher education levels are more required to work at home, and the overall demand for a home working environment is also higher.

**Conclusion:** The home-buyers' preference survey reveals that the housing unit designed based on the refined Kano model is more attractive to home-buyers. The proposed approach can help to provide important and customized decisions to design firms, housing developers, and the government for MURBs planning and strategy formulation in the post-pandemic era in China. More in-depth user surveys in other regions and investigations into the cost assessment of these strategies might be further conducted in the future.

## Introduction

The COVID-19 pandemic has caused irreparable losses to the world. According to World Health Organization, as of March 21, 2021, 2.7 million people have died worldwide (1). In addition to health issues, COVID-19 has completely paralyzed various industries or caused them millions of dollars in losses. In order to restrain the pandemic and resume work and life as soon as possible, measures such as strict protection, closed places, and social distancing have been adopted in many places (2). The method of home isolation makes the home truly a container for life (3, 4). Residential communities and buildings that are closely related to everyone's health and safety in the epidemic prevention work have become key areas for prevention and control (5).

China has a dense population and high mobility of people. Judging from the objective situation of home prevention and control implemented in the outbreak area, the number of residential home infections and the prevention and control of residential pandemics have attracted widespread attention (6). At the same time, many residential environment problems and deficiencies have gradually become prominent. According to CLV.DESIGN's “Helping the Family Fighting the Pandemic” survey launched in China in February 2020, only 20% of the 5,226 sample households collected during the pandemic were very satisfied with their home environment. Fifty six percent believed that changing houses can improve the status quo, and up to 52% of households have plans to purchase new houses (7). It can be seen that the pandemic has greatly changed the mentality of residents, and real estate developers should make adjustments and optimizations from the perspective of housing product positioning according to changes in household needs.

Inevitably, the society in the future will enter the “post-pandemic era” in which the pandemic fluctuates and lingers for a long time (5). For infectious disease disasters, residential buildings must not only meet the main needs of residents' daily lives, but also have basic epidemic prevention functions. Therefore, the design of residential buildings needs to take into account the impact of the pandemic, and must be highly adaptable to face the new normal of housing in the post-pandemic era (8, 9). In addition, China's population accounts for almost a quarter of the world's population, but in terms of urban per capita housing area, China's per capita is only 23 m^2^ (10), which is far behind many advanced countries. Regarding the choice of residential types, most residents in China can only choose relatively densely packed unit houses, and therefore multi-unit residential buildings (MURBs) has become the most common types, accounting for 90%, in the housing industry. It is more urgent to study how MURBs can better cope with the post-pandemic era, and how those professionals can control the spread of the pandemic through the design (8, 11–14).

The design standards of residential buildings and healthy buildings in the post-pandemic era have certain similarities (such as BREEAM, CASBEE, LEED, WELL, etc.), and both target human physical and mental health and well-being. The International WELL Building Institute established a task force consisting of 225 members in April 2020, which provided new resources and guidelines for the enhanced WELL building standard (15). In the face of the COVID-19 action, Fitwel also took the action of “Building Health for All” (16, 17). The action released five resources, one of which is related to buildings, that is, “using buildings to ease the spread of the virus.” The strategy for mitigating the spread of the virus is to limit body interactions, cleaning, hand washing signs, ventilation, filtration, and humidity. The theme also appears in the guide “5 Ways to Optimize Buildings for COVID-19” (18). However, the existing results are concentrated on the physical indicators of building epidemic prevention, which are not fully applicable to the design of MURBs.

Some studies have studied the connotation and influencing factors of healthy buildings (9, 19–21). Since COVID-19 occurred only 1 year ago, there is not much literature on how to respond to the pandemic in residential building design. Awada et al. (19) pointed out that more work needs to be done in the future to establish common design standards to evaluate how to design the building to support health and well-being. Megahed and Ghoneim (5) mentioned that residential buildings should increase the surrounding garden space, wider corridors, and flexibly adaptable spaces in the post-pandemic era to adapt to changing residential needs and lifestyles. Megahed and Ghoneim (2) believed that the cleaning and disinfection of the indoor environment is essential for the prevention of infection. Xu et al. (14) promoted that viruses should be avoided by cleaning the indoor surface of buildings and choosing air purification systems. Andargie et al. (22) put forward thermal conditions and indoor air quality are identified as the most important factors for overall comfort in MURBs. However, there are still limitations in the previous studies. For example, there are only few studies on the explicit design needs of MURBs in China and a complete evaluation system of MURBs is lacked. In addition, in practice, developers are often constrained by projects costs and have to make certain trade-offs of the design strategies of MURBs. Identifying the optimal strategies in the post-pandemic era to meet the preferences of occupants is a key factor in maintaining competitiveness.

The purpose of this study is to establish a set of design standards suitable to China's MURBs in the post-pandemic era, and then identifies the residents' preferences for residential design strategies through the refined Kano model. In practice, this study will make a significant contribution to the relevant industries and the government. Firstly, the proposed design strategies will encourage developers to formulate better product positioning and invest more in the development of healthy and smart housing, in order to meet the needs of their residents. Secondly, by examining these strategies, designers and architects can understand the defects of the design products in the pre-epidemic period and make good design decisions in the post-epidemic era. Thirdly, it is conducive for design firms to establish a set of standards, a housing database, and a housing evaluation system for MURBs in the post-pandemic era. Fourthly, these strategies and standards can be used by the government to formulate new MURBs design specifications and a theoretical basis. The government should also establish a cross-domain practice mechanism, integrate the concepts of green and WELL buildings, and introduce the ideas of disease prevention and control into the construction of residential communities. In addition, China's residential design itself has statutory architectural design codes as constraints. The set of design standards obtained in this study are set for user design requirements related to epidemic prevention and control without violating relevant design codes.

## Design Strategies of MURBs in the Post-Pandemic Era

### Definition of MURBs in China

**Figure 1:**
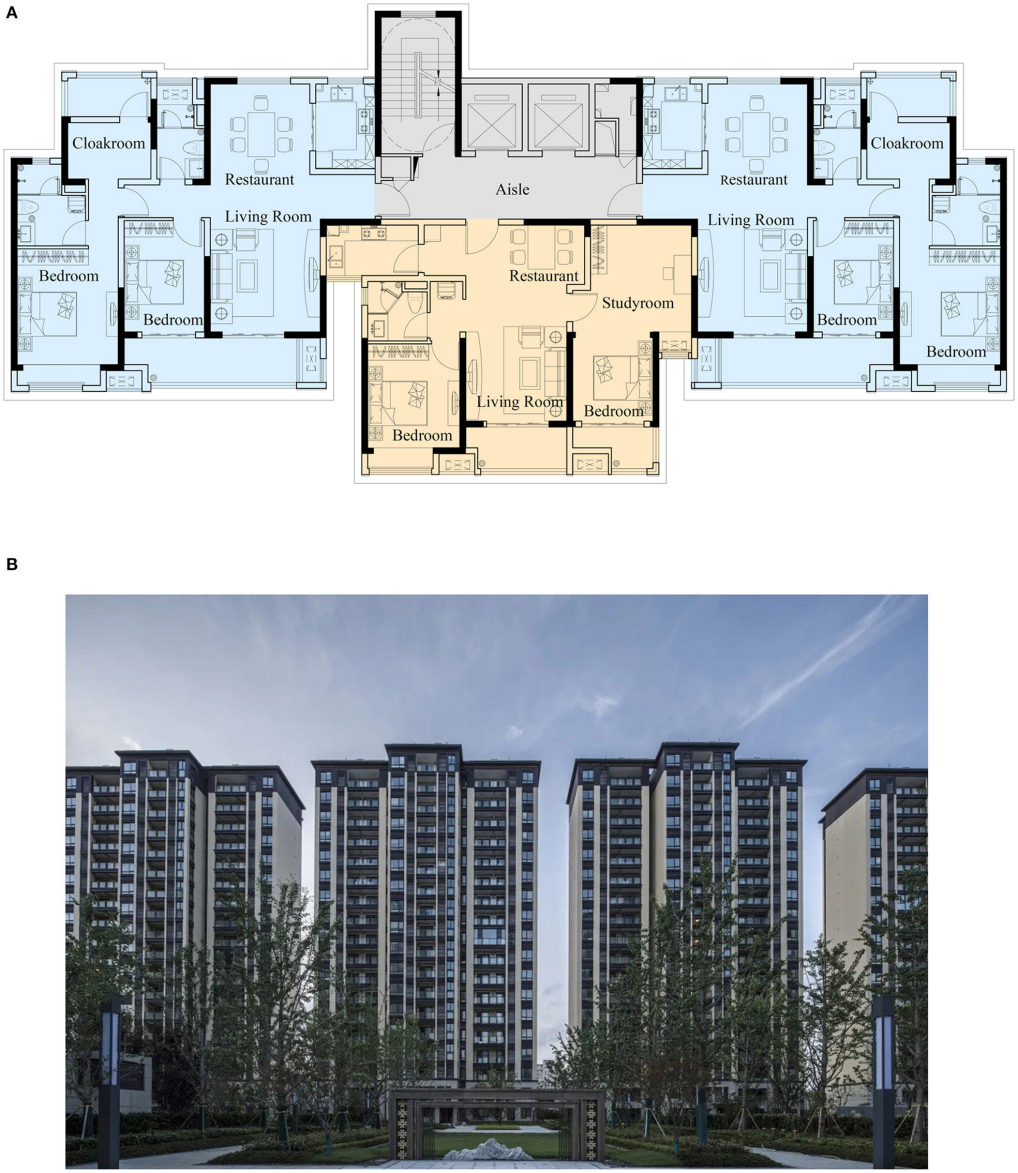
Standard floor plan and facade of a typical MURB. **(A)** Standard floor plan of a typical MURB. **(B)** Façade of a typical MURBs.

MURBs is a type of residential building in multi-story and high-rise buildings as shown in Figure 1. Usually there is only one service core per floor (formed by a combination of stairwells, elevators, halls, and corridors) for a MURB. The control area of each service core is also called a residential unit. Generally, each residential unit can be arranged with 2–4 households, and sometimes two units are arranged side by side into a slab-style unit building. Residents enter the private interior space directly from the staircase platform or waiting hall, and each resident must enter the house through public space. Most MURBs in China will be laid out in the form of residential communities, rather than a single multi-unit house appearing in the city. These communities usually contain dozens of houses, which are not crossed by arterial roads. Communities generally provide public service facilities that can meet residents' basic and social life needs.

### Development of Original Design Strategies

In order to obtain scientific and reasonable design strategies of MURBs in the post-pandemic era, this study reviewed relevant studies through search engines such as Web of Science, Google Scholor, and ScienceDirect. A total of 63 articles were found by using research methods, such as literature reviews, expert interviews, and questionnaire surveys. In terms of research content, there were 43 articles relating to building epidemic prevention design. Most of these studies focused on spatial and social distancing, ventilation, and air disinfection issues, but did not consider the users' needs to develop suitable MURBs design strategies in the post-pandemic era. Thirty one articles related to the exploration of residential health design and focused on the physical environment of buildings, such as the impact of airflow, energy-saving, lighting, and temperature control, which all have an impact on human health. Only one study discussed the implications of COVID-19 for new residential construction (3), however, it used a narrative way of describing the conceptual framing of operational spaces and design principles, and it lacked empirical research on the design strategies and verification of market demands.

This study extracted some of the design elements mentioned in the literatures. A panel of experts consisting of seven experts, including two real estate developers, two architects, one professor in the department of architecture, one professor in the department of medicine, and one infectious disease physician, was interviewed to fill the gap in the literature review. The interviews were conducted in an individual and physical face-to-face manner. These experts had conducted relevant research on built environment issues over the past few years and had experiences in participating in first-line investigations during the epidemic. In the interview process, the expert panel was requested to expand on the specific design strategies for each design element.

Viruses can be transmitted by person-to-person or person-to-object contact, as well as by droplets and aerosols in the air in confined spaces (16). Of course, disinfection can also prevent the spread of the virus to a certain extent (19). On the other hand, people locked at home during isolation must spend indoors and perform all daily activities, including eating, working, socializing, and leisure (6), so living comfort needs to be focused on in the post-pandemic era (23). In summary, this study divides the design elements of MURBs in the post-pandemic era into four dimension based on the characteristics of virus transmission and people's health needs, namely “zero contact system,” “air safety system,” “disinfection and purification system,” and “home comfort system,” as shown in Table 1, 26 design strategies are obtained according to the four dimensions through literature review and expert interview.

**Table 1:** Design system of MURBs in the post-pandemic era.

**Dimension**	**Code**	**Design strategies**	**Specific measure**
Zero-touch system	Z1	Smart access control system (5, 19, 21)	• Face recognition and mobile phone APP smart access control system at the entrance/exit of the community • Underground garage entrances and exits are equipped with automatic license plate recognition and sensorless access system • Electric induction doors (linked with face and APP unlocking functions, and optional card and fingerprint functions) are installed at the entrance hall of the underground garage and the entrance hall of the first floor • The entrance door is equipped with fingerprint recognition, password recognition and iris recognition devices, and is set to unlock and open and close automatically
	Z2	Waste collection (6, 24)	• Foot-operated trash can • Refuse sorting and collection points • Disinfection lamps and mosquito control lamps installed nearby • Hand washing and disinfection measures at the refuse collection point
	Z3	Non-contact elevator (5, 6)	• The unit door is linked to the elevator (the unit door opens with a call to the elevator, no need to press the up/down button and no need to select a floor when you enter the lift) and the lift will automatically take you to the floor where your home is located
	Z4	Infrared temperature monitoring (5)	• Thermal imaging body temperature detection and recording at all entrances and exits in the community, with automatic lever lifting after normal body temperature check
	Z5	No-touch courier reception (5)	• External drop-in and internal pick-up cabinets (with double-sided doors) or dedicated courier storage space that is well ventilated and equipped with disinfection and sterilization facilities
	Z6	Smart home (19, 21)	• The interior is equipped with a full smart home system, such as light sensor, voice control, remote control, etc • Automatic opening and closing devices are installed for each window to open and close the windows at regular intervals according to demand • Public transport aisle lighting control switch with audio-visual control
	Z7	Setting up the entrance hall (21)	• Entry gardens in each home to form a entrance hall • Separation of storage and sanitation in the entrance hall • Additional UV disinfection facilities (disinfection lamps) or disinfection solutions • Negative pressure at the entrance hall • Provision for easy cleaning devices (e.g., wash basin, ironing machine, etc.
Air safety system	A1	North-south floor plan (19)	• The floor plan is square, wide and small in depth, and there are windows or balconies facing north and south, which can maximize air convection. The living room and bedroom face south
	A2	Optimization of water supply and drainage pipes (Expert panel)	• Anti-odor floor drains • Decentralized water seal design for wet and dry areas in bathrooms • Kitchen and bathroom are designed with a non-declining slab same floor drainage system and all vertical pipes running through the floor should be isolated by pipe shafts
	A3	Ventilation system (2, 19, 25)	• Indoor split-air system and disinfection and filtration equipment for fresh air
	A4	Air quality visualization (23, 26)	• Real-time display system for indoor temperature and humidity, formaldehyde, benzene, TVOC, etc
	A5	Bathroom ventilated (21)	• Priority is given to the provision of external windows for natural ventilation in bathrooms • When there is no external window in the bathroom, direct exhaust from the same floor using water-aligned ducts, with automatic exhaust and non-return facilities
	A6	Kitchen ventilation (21)	• Elimination of the fume extraction shaft system in the kitchen and adoption of a direct outdoor system and equipment on the same level
	A7	External window bezel (Expert panel)	• In order to prevent pollution between floors, a horizontal closed baffle with a net size of not <0.6 m is used above the openings of external doors and windows, which can be freely retracted, opened when there is an epidemic and closed when there is no epidemic, so as to minimize sunlight blockage, or use closed horizontal partitions such as air conditioning partitions for floor separation
	A8	Ventilation in public areas (21)	• In lobbies, elevator halls, public walkways and stairwells, give priority to external windows for natural ventilation; when there are no external windows, install a fresh air system
Sanitization and purification system	S1	Disinfection of public areas (5)	• The elevator is regularly disinfected to enhance protection, with infrared automatic ventilation, automatic spraying disinfection or automatic activation of UV disinfection devices
	S2	Poison prevention and disinfection of the kitchen (5)	• Additional induction UV lights under the hanging cabinets • Dishwasher and other sanitary and disinfection facilities in the kitchen
	S3	Water purification at the end of the installation (6, 23)	• End-of-pipe water purification for healthier, cleaner water with fewer infections from water quality
	S4	More sunlight inside each home (21, 26)	• The spacing between buildings in the area is reasonable, and the main functional rooms in the house are well-equipped for daylighting • Open balconies are provided for each household to ensure the penetration of sunlight and ultraviolet rays
	S5	Poison prevention and disinfection of the Bathroom (5)	• An additional drainage standpipe in the bathroom, with separate drains for sewage and waste water • To provide disinfection lights or a place for disinfection solution in the bathrooms
Home comfort system	H1	Variety of living and dining rooms (5, 27)	• In living and dining rooms, Flexible furniture, partitions and other facilities can be provided to achieve space changes, which can temporarily meet the diverse functions of the current home such as office, family fitness, games, and activities
	H2	Multi-functional balcony (5, 27, 28)	• A green landscape area with plants, tables and chairs is provided in conjunction with an open balcony on the south side • A living balcony is provided for each household and an area for vegetables, food and washing and disinfection is provided on the balcony
	H3	Separate isolated master bedroom *(Expert panel)*	• Office space in the master bedroom and optimized room size • A separate bathroom in the master bedroom • Negative pressure design
	H4	Avoid indoor noise (9, 21, 27)	• Additional anti-tampering silent doors to bedrooms and study rooms
	H5	Public space for interaction (16)	• Set up a shared communication space in combination with public aisles
	H6	Community Environment (4, 5)	• Children's playground, running track, badminton court and other facilities in the community • Planting of seedlings that are rich in dust-absorbing, mosquito-repelling, anti-smoke and anti-sulfur dioxide effects

In China, most MURBs are laid out in the form of residential communities, rather than a single multi-unit building appearing in the city. Therefore, people living in MURBs are usually affected by the facilities and environment of the residential community. Therefore, the design elements in this study also include the relevant influencing factors of multi-unit residential communities.

## Application of the Refined Kano Model to Refine the Design Strategies

### Refined Kano Model

**Figure 2:**
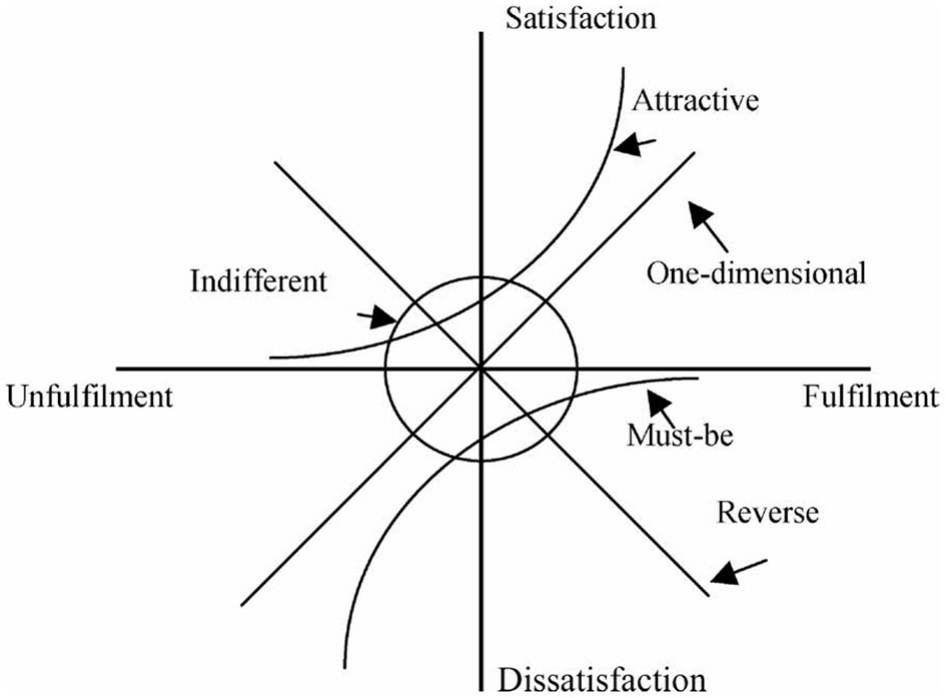
Kano model of quality attributes (29).

The Kano two-dimensional quality model (Kano model) (29) is a popular approach in the areas of product and service design and improvement because it can consider many characteristics of products or services without causing a high expense in the design process (30). The Kano model, as shown in Figure 2, divides quality attributes into five categories: (1) Attractive quality: when the elements are available, customers will be satisfied, if not, customers will not be dissatisfied; (2) One-dimensional quality: When the elements are available, the customers will be satisfied. The higher the degree, the more satisfied the customers. If not, the customers will feel dissatisfied; (3) Must-be quality: when the elements are available, the customer considers it necessary and will not be more satisfied, but when the elements are not available, the customers will be dissatisfied; (4) Indifferent quality: whether the elements are available or not, customers will not be satisfied or dissatisfied; (5) Reverse quality: When the elements are available, the customer will be dissatisfied, when not, the customer will be satisfied instead.

Conducting the Kano model requires an establishment of a questionnaire that consists of positive/functional and negative/dysfunctional questions. The respondent can answer a pair of questions in one of five different ways, “Like,” “Must-be,” “Neural,” “Live with,” and “Dislike,” for each attribute of a product (or service). The first question concerns the reaction of the customer if the product (or service) has that attribute (functional form); the second involves the reaction if the product (or service) does not have that attribute (dysfunctional form) (31). Next, the questionnaire is administered to various respondents, and each answer pair is aligned with the Kano evaluation table (32), as shown in Table 2, which can reveal each respondent's perception toward attributes of a product (or service) (33, 34). If the respondent answers, for example, “I like it that way” as regards a specific attribute from the functional side, and answers “I am neural” for the same attribute from the dysfunctional side, the combination of the question in the evaluation table will be in the “A” category, indicating that this attribute is attractive to respondent needs (35).

**Table 2:** Kano evaluation table.

**Customer requirements**	**Dysfunctional**					
		**1. Like**	**2. Must-be**	**3. Neutral**	**4. Live with**	**5. Dislike**
**Functional**	1. Like	Q	A	A	A	O
	2. Must-be	R	I	I	I	M
	3. Neutral	R	I	I	I	M
	4. Live with	R	I	I	I	M
	5. Dislike	R	R	R	R	O

However, this model has the following shortcomings: it prevents organizations from considering the importance of certain items as customers to accurately assess quality attributes. Therefore, Yang (36) altered the quality elements of Kano model into the following eight based on the degree of importance to the consumer: highly attractive and less attractive, high-value-added and low-value-added, critical and necessary, potential and care-free. In the refined Kano model, if two product requirements cannot be met at the same time, perhaps due to technical and financial constraints, the company will determine which is more crucial to customer satisfaction (31). In general, the effect of any quality characteristic on a customer's satisfaction depends on the degree to which it is valued by the customer.

The refined Kano model describes the attractive quality attributes as follows:

- Highly attractive quality: It is an effective competitive tool for the company to attract customers and can be used as a strategic quality to increase customer satisfaction.
- Less attractive quality: Since this quality factor can cause less attractiveness to customers, if the company has cost considerations, it can ignore the provision of this quality factor first.

The refined Kano model describes the one-dimensional quality attributes as follows:

- High-value-added quality: These factors can cause high customer satisfaction, thus increasing the company's revenue. Companies should be committed to providing such quality elements to customers.
- Low-value-added quality: Although these factors create less customer satisfaction, the company must continue to provide these factors to avoid increasingcustomer dissatisfaction.

The refined Kano model describes the must-be quality attributes according to the degree of consumer attention as detailed below:

- Critical quality: If such elements are necessary for the customer, the company must provide such elements.
- Necessary quality: The company should strive to provide this element to avoidcustomer dissatisfaction.

The refined Kano model describes the indifferent quality attributes as follows:

- Potential quality: Such elements may become attractive quality elements, and companies may consider providing these elements as a strategic means to attract potential customers.
- Care-free: If the company has cost considerations, it should consider not providing these elements.

**Figure 3:**
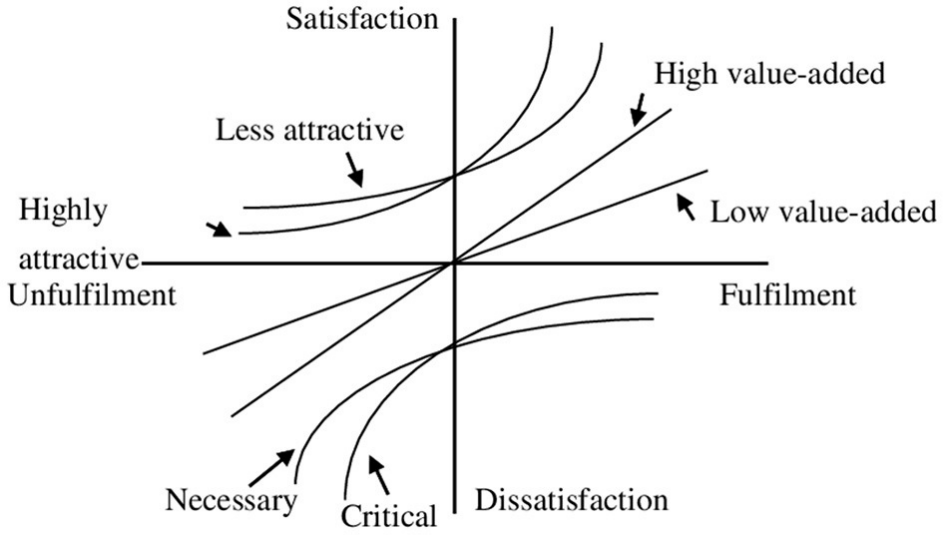
Refined Kano model of quality attributes (36).

In Figure 3, curves are used to illustrate the means of the redefinitions of quality attributes (37). Table 3 lists the redefined categories of quality attributes obtained by refined Kano model.

**Table 3:** Categories of quality attributes in refined Kano model.

**Categories of quality attributes in Kano model**	**Categories of quality attributes with high importance in refined model**	**Categories of quality attributes with low importance in refined model**
Attractive	Highly attractive	Less attractive
One-Dimensional	High value-added	Low value-added
Must be	Critical	Necessary
Indifferent	Potential	Care-free

The refined Kano model is widely used in tourism management, product design, and commercial development (38–40), and has less application in areas related to building design strategies (41). Ek and Çikis (41) adopted the Kano model into the cases of architecture to explore architectural design quality for mass-housing projects. The result suggested that the Kano model can demonstrate the flexibility of interpretation and this flexibility can guide architects in improving the future spatial-programmes of the building projects. Juan et al. (35) employed the Kano model and a customer satisfaction matrix to evaluate professional designers' and general users' satisfaction, preferences, and acceptability of intelligent green building design strategies. The result revealed that the proposed approach could be a useful tool to explore similarities and discrepancies of strategy preferences between designers and users, and these findings could effectively decrease the communication gap for future building design. Juan et al. (42) further applied the Kano model and the quality function deployment (QFD) method to identify design strategies for public housing projects. The result indicated that the systematic methods can uncover design and planning strategies for public housing, serving as a guide and reference for future designing. According to the above studies, the Kano model has great potential, as the main research method, to develop architectural design strategies. Therefore, after the proper modification of the Kano model, this study integrates the design strategies of MURBs in the post-pandemic era with the refined Kano model to explore the perceived importance and satisfaction of the occupants, and to identify the critical key aspects of design strategies that should be prioritized for improvement.

### Questionnaire Design

The indicators of the questionnaire design of this study are derived from the results obtained by the experts in Table 1. The questionnaire design consists of three parts: the first part is personal anonymous information, including the sex, age, education, and residence of the tested person. The second part is a Kano-based questionnaire with 26 design strategies. The third part is the importance evaluation of the 26 strategies, which are answered on a five-point Likert scale, with 1 being “very unimportant” and 5 being “very important.”

### Data Collection and Sampling Structure

The research team chose to conduct the refined Kano-based questionnaires in Jiangsu Province. The reasons for choosing Jiangsu Province as the research object are as follows: first, Jiangsu Province is one of the most economically active provinces in China and consists of 13 prefecture-level cities. The Yangtze River Delta urban agglomeration formed by Shanghai, Zhejiang, and Anhui has become one of the six world-class city group. Among them, Jiangsu has a well-developed construction industry, and the number of construction projects has consistently ranked first in China; second, as of the end of 2019, Jiangsu Province has a permanent population of 80.7 million, which is the most densely populated province in China; third, Jiangsu Province belongs to the East Asian monsoon climate zone and is in a subtropical and warm temperate climate. In the transition zone, the climate has both the characteristics of the south and the north. Therefore, it is reasonable to use it as a representative of analyzing the residents' needs of MURBs in China in the post-pandemic era.

The official survey was launched in July 2020 and was conducted in three cities located in Jiangsu Province: Huai'an, Yangzhou and Suzhou, representing the northern, central and southern regions of Jiangsu Province, respectively. The survey was divided into two stages: small-scale testing and random sampling survey. The small-scale test is to ensure the validity of the questionnaire. Before the questionnaire was formally issued, 10 residents living in multi-unit houses in Yangzhou were pre-tested. Based on the feedback, some improvements were made, which resulted in the final questionnaire. In the random sample survey stage, both online surveys and on-site surveys are conducted. On-site surveys can make up for the lack of sample characteristics of the online survey form by targeting the imbalance in the number and quality of urban residents.

A total of 395 questionnaires were distributed and a total of 376 valid questionnaires were received. Data analysis of the questionnaire was mainly carried out by statistical software SPSS 26.0. Reliability analysis showed that values of Cronbach's alpha are 0.895, 0.932, and 0.811 for positive/functional questions, negative/dysfunctional questions, and the importance of questions, respectively. The result also revealed that the reliability of the questionnaire in this study is relatively high, which was suitable for subsequent analysis (43). After excluding all kinds of interference and invalid questionnaires, 324 of them were adopted for the analysis. As the population in the three regions of Jiangsu Province (northern, central and southern) accounted for 37.82, 20.80, and 41.38% of the total population of Jiangsu Province, respectively (44), and the final sample sizes in the northern, central and southern regions of Jiangsu Province were 112, 79, and 133 respectively. The final results of the survey sample were generally consistent with these proportions. The sample structure is shown in Table 4. All the interviewees are residents living in MURBs, of which 57.10% are female respondents, slightly more than males. The number of respondents between the ages of 36–49 years old and a college degree accounted for the largest number of respondents.

**Table 4:** Demographic data of questionnaire.

**Characteristics**		**Frequency**	**Percentage (%)**
Place of residence	Northern	112	34.57
	Middle	79	24.38
	Southern	133	41.05
Gender	Male	139	42.90
	Female	185	57.10
Age	18–35	86	26.54
	36–49	151	46.60
	50–64	59	18.21
	≥65	28	8.64
Educational level	High school or below	86	26.54
	College	189	58.33
	Graduate school or above	49	15.12

## Results

According to the judgment rules of the Kano model in Table 2 and the defined categories of the refined Kano model in Table 3, the results of the 26 questions (strategy items) are shown in Table 5. All the strategy items are attributed to attractiveness, one-dimensional and must-be quality. There is no reversal and indifferent strategy item, which proves that the impact of the aforementioned standards on residents is positive and effective. Among them, the proportions of attractive, one-dimensional and must-be strategy item are 50, 26.92, and 23.08%, respectively. There are thirteen strategy items in the attractive quality attribute including: H3, H2, A3, Z6, H1, H5, S1, S3, Z3, A7, A4, Z5, and Z4. It shows that the provision of these 13 design strategies can significantly increase homebuyer satisfaction and can bring them the surprise of being delighted with the completed property. In contrast, if the MURBs does not meet these design criteria or is inadequate it does not cause homebuyer dissatisfaction.

**Table 5:** Result of the refined Kano model.

**Kano model**	**Code**	**Design strategies**	**Refined Kano model**		
			**Importance ranking**	**Importance**	**Quality attributes**
Attractive quality	H3	Separate isolated master bedroom	7	4.11	Highly attractive quality
	H2	Multi-functional balcony	8	4.07	
	A3	Ventilation system	10	4.00	
	Z6	Smart home	13	3.93	
	H1	Variety of living and dining rooms	14	3.89	
	H5	Public space for interaction	15	3.84	
	S1	Disinfection of public areas	18	3.56	Less attractive quality
	S3	Water purification at the end of the installation	20	3.44	
	Z3	Non-contact elevator	22	3.41	
	A7	External window bezel	22	3.39	
	A4	Air quality visualization	23	3.37	
	Z5	No-touch courier reception	25	3.30	
	Z4	Infrared temperature monitoring	26	3.22	
One-Dimensional quality	S4	More sunlight inside each home	1	4.48	High-Value-Added quality
	S5	Poison prevention and disinfection of the bathroom	6	4.15	
	Z7	Setting up the entrance hall	9	4.06	
	S2	Poison prevention and disinfection of the kitchen	11	3.96	
	H6	Community environment	12	3.94	
	H4	Avoid indoor noise	16	3.70	Low-Value-Added quality
	Z2	Waste collection	24	3.33	
Must-be quality	A1	North-south floor plan	2	4.37	Critical quality
	A5	Bathroom ventilated	3	4.33	
	A6	Kitchen ventilation	4	4.30	
	A2	Optimization of water supply and drainage pipes	5	4.22	
	Z1	Smart access control system	17	3.59	Necessary quality
	A8	Ventilation in public areas	19	3.50	

The seven design strategies of S4, S5, Z7, S2, H6, H4, and Z2 are categorized as one-dimensional quality attribute. That is, there is a linear relationship between performance and satisfaction of the strategy item. Respondents generally felt that the more supply of these design strategies, the more satisfied they are, and the less satisfied they are when the supply is less. Less or more here sometimes refers not only to quantity, but also to degree or effectiveness. For example, the design strategy of “H4-more sunlight and daylight” and “H6-community environment,” for most interviewees, are considered that the better the performance, the better the satisfaction and the sense of security.

A1, A5, A6, A2, Z1, and A8, a total of 6 design strategies, are classified as must-be quality attributes. Respondents believe that MURBs must have the above elements, otherwise it will lead to unsatisfactory reactions. However, further increase of these qualities cannot effectively improve the satisfaction of residents. In other words, this type of quality attribute has a strong influence on the low satisfaction part of the occupants, but has no obvious influence on the high satisfaction part. In terms of design management, it means that this type of quality attribute is a basic essential feature, designers must maintain a certain level of such design elements, but do not need to pursue high standards leading to a waste of design resources.

This study further divides the traditional Kano attribute categories into different categories based on the importance of strategy items. The first step is to calculate the average of the importance of each item, and then calculate the overall importance of the item. Next, comparing the two values and identify those items which are greater than the overall average are considered to be high importance, and those less than the overall average are considered to be low importance. The overall average number of 26 strategy items in the questionnaire is 3.83. Afterwards the Kano model can be transformed into the refined Kano model according to the importance comparison, as shown in Table 5.

As shown in Table 5, H3, H2, A3, Z6, H1, and H5 are classified as highly attractive quality, which is an emotional attribute that is very attractive to occupants, and is an important competitive weapon for multi-unit residential products. S1, S3, Z3, A7, A4, Z5, and Z4 are classified as less attractive quality, indicating that although they can attract occupants, the effect is not significant. When the cost is insufficient in the design proposal, the needs of these strategies can be ignored. S4, S5, Z7, S2, and H6 are classified as high-value-added quality qualities which should make a significant contribution to the satisfaction of the respondents and every effort should be made to increase the availability of these strategies in order to enhance the preference of the occupants. H4 and Z2 are classified as low-value-added quality attribute, which proves that they do not make a great contribution to the occupants. Although the value of these strategies are slightly lower, they still cannot be ignored. A1, A5, A6, and A2 are the critical quality classifications, which means they are considered necessary by the respondents. These needs to satisfy the occupants should be paid attention to in the future design. Z1 and A8 are categorized as the necessary quality attribute, and although they are still must-be quality, they are less important. Real estate developers only need to provide appropriate specifications of design, but they must also avoid dissatisfaction with buyers due to low specifications.

It is worth noting that according to face-to-face interviews with respondents, many of them generally believe that in the post-pandemic era, once quarantined, people are forced to stay indoors for longer, so they prefer paying more attention to the anti-epidemic function of living in the indoor space. Design strategies such as H3, H2, H1, and H5 can enhance the comfort of their living space. In addition, the design strategies such as Z3, A7, A4, Z5, and Z4 are all emerging technologies that are costly and will drive up the price of housing. For most Chinese homebuyers, particularly those on low and middle incomes, their lack of understandings of these technologies has led to a significant reduction in the importance evaluation. However, these strategies are also part of the attractive attributes that can add a sense of surprise for occupants when these design items are available. In the future, if real estate developers have the funding to include these low attractive strategies, potential homebuyers may improve their preference and willingness to purchase the housing.

In summary, the results have shown that the refined Kano model can indeed effectively assist designers and real estate developers to understand the real preferences of homebuyers. The abovementioned strategies, such as 6 items of highly attractive attribute, 5 items of high-added-value attribute, and 4 items of critical attribute should be fully met by developers and they can become important marketing strategies in the post-pandemic era. In addition to the 7 items with lowly attractive attribute that can be discarded when project funds are not available, developers should also need to provide the remaining 19 design strategies and evaluate their cost-benefit analysis to reconsider their implementation priority.

## Discussion

### Result Verification: Home-Buyers' Preference Survey

**Figure 4:**
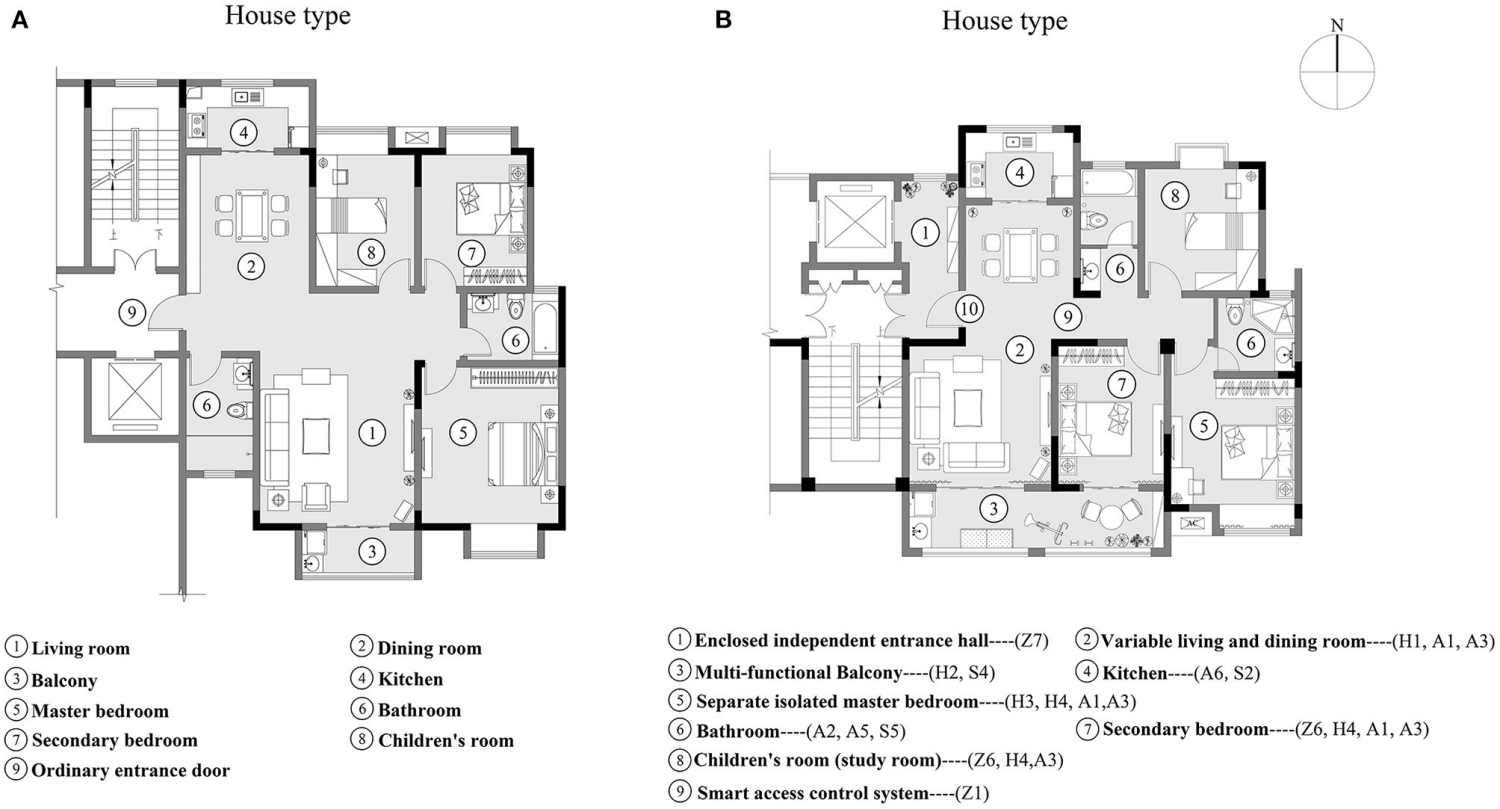
Two housing units [Type **(A)** and Type **(B)**] for preference survey.

In order to verify the reliability of the results of the refined Kano model, the research team cooperated with a real estate developer to provide two types of housing units in the initial planning and design stage, as shown in Figure 4. One was for an ordinary unit (Type A), which did not adopt the results of the refined Kano model, and the other was the unit (Type B) that was designed, based on the results of the refined Kano model. Both housing units have an area of 115 m^2^ and the housing prices are also the same. The respondents were potential home-buyers and were asked to choose which unit they were willing to buy and live in. The research team launched a questionnaire in the Jiangsu Province in June 2021 by means of the Internet. While distributing the questionnaire, the research team also provided a 5 min pre-recorded online video introducing design features of Type A and Type B, so that the respondents could fully understand the differences between the two design units. A total of 62 valid questionnaires were collected. Among them, 49 respondents selected Type B, which accounted for 79.03%, and a total of 13 respondents selected Type A, which accounted for 20.97%. The results revealed that Type B, which is based on the results of the refined Kano model, is more attractive to homebuyers.

### Comparison With Existing Studies

Comparing the results of this study with existing research on residential buildings in the context of COVID-19, there are some similarities and differences that can be discussed. In terms of similarities, the design strategies that fall into the attributes of “Highly attractive quality,” “High-value-added quality,” and “Critical quality” are generally in line with the considerations put forward in international research. In other words, in the COVID-19 epidemic, these strategies will not be different due to differences in national conditions, living habits and residential behaviors.

There are some anti-epidemic design strategies that are considered effective by many studies, but the results of this study are relatively insignificant. For example, many studies have mentioned the importance of intelligent strategies for epidemic prevention and control (19, 23). However, most of these strategies (such as Z3, A4, Z5, and Z4) belong to the attribute of “Less attractive quality.” It is speculated that the reason may be related to the lack of experience in the use of intelligent technologies by most interviewees, and they are worried that these technologies will increase housing prices and reduce the importance of evaluation. It is suggested that the Chinese government can provide cost subsidies for these intelligent anti-epidemic strategies in the policy of housing development. Housing developers can also provide more product experiences and cost-benefit evaluations to increase incentives for future project applications.

It is worth mentioning that there are two strategies that fall into the attribute of “Low-value-added quality,” namely “H4-Avoid indoor noise” and “Z2-Waste collection.” Although the importance of these two design strategies is slightly lower, they cannot be ignored. Regarding H4, Tleuken et al. (23) believe that in the post-epidemic era, indoor sound insulation design should be adjusted to improve private space and psychological comfort. In China, many people were forced to stay at home for several months when the city was closed due to COVID-19. The balance between remote office and family life has become an important issue. Improper noise interference can affect work efficiency and restrict family activities. At present, most architects and developers do not pay special attention to indoor noise prevention in the design of MURBs. In the future, it should be valued and integrated into indoor sound insulation design, including the selection and design of sound insulation materials.

The same situation also occurs in the strategy of “Z2-Waste collection.” Many studies have pointed out that in the post-epidemic era, the planning of waste collection stations is very important. In particular, residents should keep distance and avoid contact with surfaces to avoid the spread of the virus (24). Although the results of the survey show that the strategy has a certain degree of importance, the interviewees generally ignore the impact of the strategy. In other words, the public still needs to strengthen and enhance their awareness of epidemic prevention, and housing developers should also play a more active role, helping good strategies be planned and implemented.

### Implication for Design Practices and Policies

This study summarizes the relationship between the respondents' place of residence, gender, age, education variables and their preference for a design strategy, as shown in Table 6. From the concept of the refined Kano model, the strategy with a “highly attractive” attribute can be defined as a differentiation strategy. Take the place of residence, as an example, the residents in the northern area prefer Z7, while Z6 (the smart home) and H2 (the multi-functional balcony) are “less attractive.” In other words, these two design strategies are generally less attractive to residents in the northern regions. In terms of gender, it is obvious from Table 4 that females' preferences of design strategies are relatively pragmatic and conservative. Some males believe that design strategies that can increase the selling points are the basic needs of females. Males also generally believe that it is necessary to provide a space for public communication in planning and design in the post-epidemic era (H5). Different age groups also reflect different design strategy preferences. For example, retirees over 65 years old are less likely to work at home. Therefore, the separate, isolated master bedroom with office space (H3) is obviously less needed. In terms of education variables, those with a higher educational level generally do not need Z6 and H2, but they have higher expectations with respect to noise prevention (H4).

**Table 6:** Relationship between different variables and preferences of design strategies.

**Variables**		**Highly attractive quality**	**High-value-added quality**	**Critical quality**
Area	Northern	A3, H1, H3, H5	Z7, S2, S4, S5, H6	A1, A2, A5, A6
	Middle	Z6, A3, H1, H2, H5	S2, S4, S5, H6	A1, A2, A5, A6
	Southern	Z6, A3,H1, H2, H5	S2, S4, H6	A1, A2, A5, A6
Gender	Male	Z6, A3, H1, H2, H3	Z7, S2, S4, S5, H6	A1, A2, A5, A6, H5
	Female	H2, H3	S2, S4, S5,H6	Z6, Z7, A1, A2, A3, A5, A6, H1
Age	18–35	Z6, A3, H1, H2, H3, H5	Z7, S2, S4, S5, H6	A1, A2, A5, A6
	36–49	Z6, A3, H1, H2, H3	Z7, S2, S4, S5, H6	A1, A2, A5, A6
	50–64	Z6, A3, H3, H5	Z7, S2, S4, S5, H6	A1, A2, A5, A6
	≥65	Z6, A3, H1, H5	Z7, S2, S4, S5, H6	A1, A2, A5, A6
Education	High school or below	Z6, A3, H1, H2, H5	Z7, S2, S4, S5, H6	A1, A2, A5, A6
	College	Z6, A3, H1, H2, H5	Z7, S2, S4, S5, H6	A1, A2, A5, A6
	Graduate school or above	A3, H1, H3, H5	Z7, S2, S4, S5, H4, H6	A1, A2, A5, A6

According to the analysis results of Table 6, developers and designers in the future can make customized strategic positioning, in order to target and meet the needs of their customers. Assuming that the target customers in the future are young men with a higher level of education, in addition to satisfying their basic needs, the design should try to increase the charm of the strategies, such as the provision of smart home technologies (Z6), a fresh ventilation system (A3), a variety of living and dining rooms (H1), a multi-functional balcony (H2), a separate isolated master bedroom (H3), the prevention of indoor noise (H4), and a public space for interaction (H5).

From the perspective of industrial development, the new concept of healthy housing in the post-epidemic era must also be integrated into housing development policies, and healthy housing should be integrated with housing design, construction, and operation to improve the integrity of the building life cycle. For housing developers, the design strategies obtained by this study can be used as the inspection standards for the subsequent house delivery, as well as the project management process and control points to enhance the competitiveness of the enterprise. On the other hand, the developers can combine their own characteristics, residents' concerns, and technical cost-effectiveness to formulate corresponding residential unique systems, reflecting the residents' higher demand for residential performance. For example, in the development of healthy and smart residential products, developers can conduct special research on integrating bathrooms and kitchens, adding sterilization and disinfection equipment, and formulating corresponding solutions for customer complaints and dissatisfaction.

Designers and design firms should optimize their design strategies further according to the new residential needs, and take the design of healthy and smart housing as the added value of future residential building products. Such strategic adjustments will help to form differentiated strategies, to accumulate relevant design experience and technology, to improve a company's reputation, and finally, to enhance the competitiveness of business operations in the post-pandemic era. Finally, the government should actively formulate new residential building design standards and codes adapted to the post-epidemic era. At the same time, the government should also establish a cross-domain practice mechanism, integrate the concepts of green and WELL buildings, and introduce the ideas of disease prevention and control into the construction of residential communities.

## Conclusion

The current COVID-19 pandemic is one of the greatest challenges facing the world's health and economy, but COVID-19 is not the first pandemic, nor the largest pandemic, and may not be the final pandemic. The impact of this pandemic on the entire society will be very far-reaching. In China, the dense urban population and high mobility increase the risk of outbreaks of infectious diseases. As the basic spatial unit for epidemic prevention and control, urban residences can build an effective control and defense line for epidemic prevention. However, at present, the problems of China's largest stock of MURBs have arouse widespread thinking among governments and scholars, what kind of housing can truly “protect” us? From the perspective of epidemic prevention, health, and comfort, it can be seen that occupants need all-round houses with better system performance, more practical space functions, and better technical components. It is also believed that healthy buildings with super immunity will obtain greater competitive advantages in the housing supply market in the future.

This study summarizes the four dimensions and 26 design strategies of MURBs in China during the post-pandemic era. After analyzing the results of refined Kano questionnaires, 13 attractive, 7 one-dimensional, and 6 must-be quality attributes were obtained, and 6 highly attractive, 5 high-value-added and 4 critical quality attributes were further extracted. In future MURBs projects, housing developers should pay more attention to these design strategies and formulate marketing solutions that are based on actual economic conditions, and they should also propose rationalized and fully-decorated delivery suggestions for their products to improve their core competitiveness. Furthermore, other types of public buildings, not only the residential projects, will be more focused on the application of new technologies. Therefore, designers should re-adapt to the application capabilities of new technology in the post-epidemic era, according to the changes in user needs. Design firms need to further increase the design management and technical training of their staff. Most importantly, they should establish a set of MURBs standards for different housing types in the post-pandemic era, so that these design firms can actively participate in the development of industry standards and technical specifications, in order to continually enhance their influence in the industry.

This study conducted a preliminary exploration based on the residential needs of urban inhabitants in Jiangsu Province in the post-pandemic era. Jiangsu is one of the provinces with the fastest economic growth in China and is regarded as a demonstration area for the coordinated development of economic, social and environmental systems. Therefore, the results can provide relevant and meaningful insights for other similar countries and regions. However, this study also has certain limitations. First of all, due to the limitation of sample size, only the research sample of Jiangsu Province of China is considered. In other regions of China, due to their specific climate and economic conditions, people may have different preferences in choosing residential design strategies. It is recommended that more in-depth research in other regions should be conducted in the future. Secondly, due to differences in demographic characteristics such as age, gender, education level, and occupation, different user groups have different preferences for MURBs design features. There are still many topics worth studying in the future post-epidemic era. For example, researchers can consider further the cost optimization of these design strategies, in order to provide building developers and residents with a premium assessment. The new design requirements in the post-epidemic era also require the re-training in relevant design techniques and tools of designers, the redefinition of design products by design firms and investment budgets, and the performance evaluation of various design strategies by developers. Finally, the trend of building intelligence and informatization will become more prominent in the post-epidemic era. Another important direction for future research is the integrated study of BIM and virtual reality technologies to support and manage user experiences and needs in the post-epidemic era.

## Data Availability Statement

The raw data supporting the conclusions of this article will be made available by the authors, without undue reservation.

## Ethics Statement

Written informed consent was obtained from the individual(s) for the publication of any potentially identifiable images or data included in this article.

## Author Contributions

All authors listed have made a substantial, direct and intellectual contribution to the work, and approved it for publication.

## Funding

The authors are grateful for the funding supported by the Taiwan Building Technology Center from The Featured Areas Research Center Program within the framework of the Higher Education Sprout Project by the Ministry of Education (MOE) in Taiwan (Grant No. 110P011, 2021).

## Conflict of Interest

The authors declare that the research was conducted in the absence of any commercial or financial relationships that could be construed as a potential conflict of interest.

## Publisher's Note

All claims expressed in this article are solely those of the authors and do not necessarily represent those of their affiliated organizations, or those of the publisher, the editors and the reviewers. Any product that may be evaluated in this article, or claim that may be made by its manufacturer, is not guaranteed or endorsed by the publisher.
